# Beneficial Effects of Melatonin on Apolipoprotein-E Knockout Mice by Morphological and ^18^F-FDG PET/CT Assessments

**DOI:** 10.3390/ijms21082920

**Published:** 2020-04-22

**Authors:** Lorenzo Nardo, Rita Rezzani, Luca Facchetti, Gaia Favero, Caterina Franco, Yasser Gaber Abdelhafez, Ramsey Derek Badawi, Michele Guindani, Youngho Seo, Miguel Pampaloni

**Affiliations:** 1 Department of Radiology, University of California, Davis, CA 97817, USA; lnardo@ucdavis.edu (L.N.); yabdelhafez@ucdavis.edu (Y.G.A.); rdbadawi@ucdavis.edu (R.D.B.); 2 Anatomy and Physiopathology Division, Department of Clinical and Experimental Sciences, University of Brescia, 25123 Brescia, Italy; gaia.favero@unibs.it (G.F.); c.franco@studenti.unibs.it (C.F.); 3 Interdipartimental University Center of Research “Adaptation and Regeneration of Tissues and Organs (ARTO)”, University of Brescia, 25123 Brescia, Italy; 4 Department of Radiology, University of Brescia, 25123 Brescia, Italy; Facchettil@gmail.com; 5 Nuclear Medicine Unit, South Egypt Cancer Institute, Assiut University, Assiut, PO 71516, Egypt; 6 Department of Statistics, University of California, Irvine, CA 92697, USA; Mguindan@uci.edu; 7 Department of Radiology and Biomedical Imaging, University of California, San Francisco, CA 94158, USA; youngho.seo@ucsf.edu (Y.S.); miguel.pampaloni@ucsf.edu (M.P.)

**Keywords:** atherosclerosis, melatonin, macrophage polarization, perivascular brown adipose tissue, molecular imaging, PET/CT

## Abstract

Atherosclerosis represents one of the main risk factors for the development of cardiovascular diseases. Their etiologies have been studied in recent years in order to better define therapeutic targets for intervention and to identify diagnostic methods. Two different subtypes of macrophages, M1 and M2, have been described in physiological conditions. They can also be found in the atherosclerotic process, where they both have opposite roles in disease progression. Perivascular brown adipose tissue is also involved in inflammation and endothelial damage. In this work, we provide insights into the protective role of melatonin in the atherosclerotic process by morphological and ^18^F-FDG-PET/CT analyses. In particular, we examined the effects of melatonin on pathways that are linked to atherosclerosis development. We showed that melatonin, by suppressing M1 activity, reduced inflammation and directed macrophage polarization toward the M2 macrophage subtype. Moreover, melatonin preserved the activity of perivascular brown adipose tissue. In addition, ^18^F-FDG uptake is very high in mice treated with melatonin, confirming that other factors may alter ^18^F-FDG distribution. In conclusion, we showed that melatonin affects inflammatory pathways that have been linked to atherosclerosis, assessed the relationships of the ^18^F-FDG PET/CT parameters with macrophage markers and the production of their cytokines, which that have been defined by morphological evaluations.

## 1. Introduction

Atherosclerosis has long been known as the main cause of cardiovascular diseases (CVDs) and only recently, more attention has been paid to the many factors that can contribute to the development and progression of this process itself [1,2]. Atherosclerosis has a multifactorial etiology, but it shows similar mechanisms such as chronic inflammation and immune activation [3,4]. In particular, inflammation also activates both innate and adaptive immune responses involving monocytes/macrophages [5]. M1 macrophages promote plaque development, while M2 macrophages promote tissue repair and plaque stabilization [6]. More recently, Barrett reported that M1 macrophages are involved in initiating and sustaining inflammation, while M2 cells are implicated in inflammation resolution [7]. M2 macrophage enrichment in plaque regression is consistent with the data that M1 cells are pro-atherogenic and promote an unstable plaque [7]. Moreover, it is important to remember that these cells produce several cytokines [8].

Another fundamental risk factor for atherosclerosis is perivascular adipose tissue [3,9]. Perivascular adipose tissue alterations lead to vascular endothelial and smooth muscle cell dysfunctions. Moreover, perivascular adipose tissue has characteristics that resemble both brown adipose tissue (BAT) and white adipose tissue (WAT).

For the reasons reported above, many cardiovascular studies have evaluated the role of adipose tissue in atherosclerosis progression, focusing on agents that can modulate the inflammatory and immune pathways. Two of the main scientific points that should be stressed are: (1) most of the trigger causes leading to atherosclerosis are modifiable, so it is important to find novel therapeutic interventions with a wider range of action [10], and (2) the current timing of diagnosis and intervention is not able to assure proper management of the disease. Thus, it is important to evaluate and study new methods that can recognize and early identify the evolution of this pathologic process [2].

Regarding the first point, many recent studies have focused on how to reduce the progression of the atherosclerotic process. Among them, particular attention has been paid to melatonin (MLT). MLT, an indoleamine physiologically produced by the pineal gland mainly during the night as well by other organs, is often available as a dietary supplement [11,12,13]. It is involved in several functions and has powerful antioxidant properties [14,15,16,17]. MLT also improves endothelial functions and possesses anti-inflammatory properties [18]. Moreover, recent evidence has confirmed the role of MLT as a new agent for atherosclerotic pharmacotherapy.

According to the findings of Høilund-Carlsen et al. and our considerations, it is important that innovation in diagnostics supports renewal therapies [19]. In particular, the use of ^18^F-fluorodeoxyglucose (^18^F-FDG) positron emission tomography (PET) as an imaging modality for studying inflammation in atherosclerosis is a new, but not well-defined measure of atherosclerotic plaque [20,21,22]. It has been identified as a potential new “gold standard” approach for studying early-stage atherosclerosis considering its non-invasiveness. In fact, this technique allows an early grading of the disease when it may still be susceptible to therapy [19]. Furthermore, ^18^F-FDG is considered as a good indicator of aerobic glycolysis in tumor tissue [23].

Considering the findings above reported, the objectives of this study were (1) to morphologically confirm and better evaluate the role of MLT in modulating perivascular brown adipose tissue of apolipoprotein-E knockout (ApoE^-/-^) mice, a known model of atherosclerosis; and (2) to compare the morphological evaluations with results obtained with ^18^F-FDG PET combined with computed tomography (PET/CT) in order to better define the possible use of these analyses in the detection of early-stage atherosclerosis and its progression.

## 2. Results

All animals in both experimental groups were weighed at the beginning and at the end of the study. There were no statistically significant differences in body weight between the two groups at any of the weekly measurements across the study duration. Body weight measurements of the control and MLT-treated groups were 18.0 ± 1.3 g and 18.9 ± 1.5 g at the first time point (*p* = 0.2) and 38.5 ± 4.0 g and 41.8 ± 4.4 g at the last time point (*p* = 0.13), respectively. In both cases, the last time evaluation showed significantly increased values as a result of the atherogenic high fat “Western” diet with which both experimental groups were treated.

### 2.1. Melatonin Induces Browning of Periaortic Adipose Tissue

**Figure 1:**
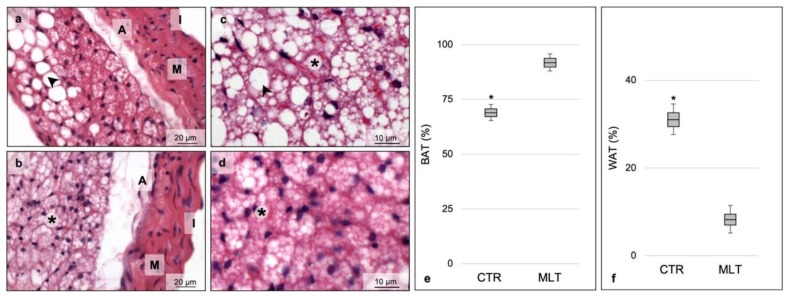
Morphometrical evaluation. Photomicrographs of the aorta from the control (**a**,**c**) and MLT-treated (**b**,**d**) mice. Each panel shows a full-field image at 400× (**a**,**b**) (scale bars: 20 µm) and at 1000× (**c**,**d**) (scale bars: 10 µm). Control group aorta showed larger areas of WAT, characterized by white lipid drop, with a minimum presence of multilocular brown adipocytes. In addition, ApoE^-/-^ mice aorta is also characterized by a disarrangement of normal vascular structure (**a**). On the contrary, MLT-treated mice showed a higher presence of BAT, with a significant reduction of white adipocyte infiltration and are also characterized by relatively unremarkable changes in vascular cytoarchitecture and organization (**b**). Photomicrographs of perivascular adipose tissue from the control (**c**) and from treated (**d**) mice. Graphs summarize the morphometrical analyses of the percentage per area of periaortic BAT (**e**) and WAT (**f**) obtained evaluating, for each experimental animal, fifteen non-overlapping fields with the same area. Statistical analyses comparing multiple continuous outcomes were performed using one-way analyses of variance test corrected by Bonferroni for morphometrical evaluations. Continuous variables are summarized as means ± standard deviation. Error bars represent the 95% confidence interval around the mean; * indicates the level of significance, *p* ≤ 0.05; black asterisk indicates BAT, brown adipose tissue; black arrowhead indicates WAT, white adipose tissue; I, tunica intima; M, tunica media; A, tunica adventitia; CTR, control group; MLT, mice treated with melatonin.

Hematoxylin-eosin staining showed the distribution of white and brown adipocytes in the periaortic adipose tissue of both experimental groups. According to Manieri and colleagues, in fact, it is possible to find a correspondence between the morphological considerations and the results obtained from the immunohistochemical evaluations of some proteins used as markers of specific subpopulation of adipocytes [24]. In the present study, the control group presented larger areas of periaortic adipose tissue with characteristics of white adipocytes, characterized by unilocular lipid-laden drops, with a minimum presence of multilocular BAT adipocytes (Figure 1a). In contrast, the group of MLT-treated mice showed a higher presence of cells with characteristic features of brown adipocytes, namely multilocular lipid droplets, with a significant reduction of white adipocyte infiltration (Figure 1b). These data suggest that MLT treatment induced a shift in periaortic adipose tissue composition from primarily one of white adipocytes to primarily brown adipocytes.

Figure 1c,d show, at higher magnification, the different organization of the periaortic adipose tissue with respect to the control and MLT-treated groups. The morphometrical analyses of periaortic BAT (Figure 1e) and WAT (Figure 1f) confirmed the previously reported observations.

It was also possible to recognize morphological alterations in the aortic wall in the control group (Figure 1a) due to the presence of atherosclerotic-related lesions. The aortic structural disarrangement was not evident in the MLT-treated mice (Figure 1b).

### 2.2. Aortic Inflammatory State

**Figure 2:**
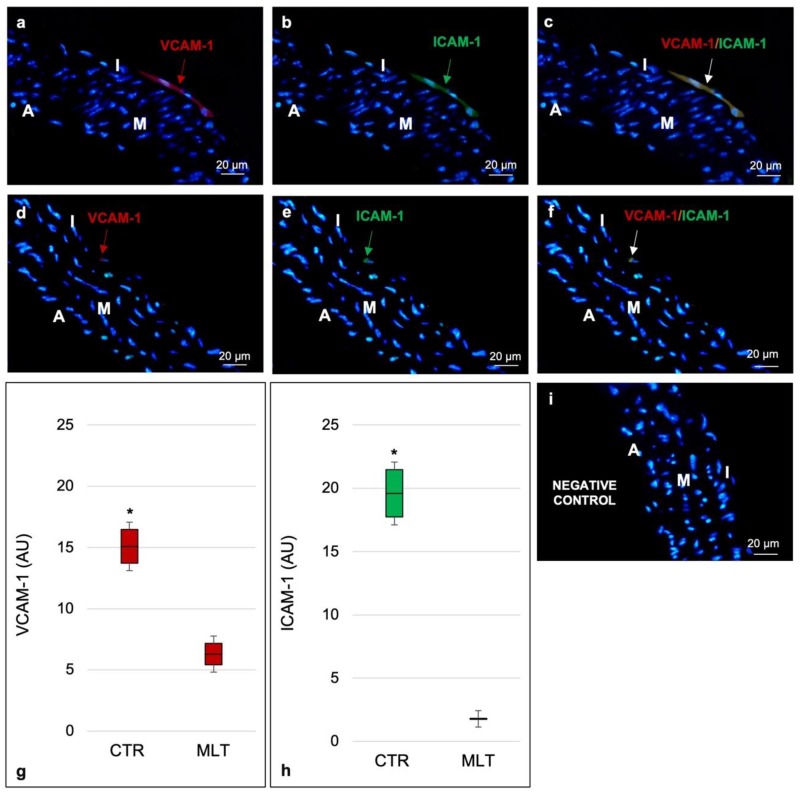
Assessment of the inflammatory state. Labelling of the aorta’s wall with VCAM-1 (identified in red staining, panels (**a**) and (**d**) and ICAM-1 (identified in green staining, panels (**b**) and (**e**) considering them as general markers of the inflammatory state. Each panel shows a full-field image at 400× (scale bars: 20 µm). Double immunofluorescence photomicrograph of the control mice shows a moderate expression of both adhesion proteins (merged expression reported in panel (**c**). In contrast, the double immunofluorescence photomicrograph of ApoE^-/-^ mice treated with melatonin highlights, for the same inflammatory markers, an absent/very weak positivity at the tunica intima level (merged expression reported in panel (**f**). Graphs summarize, as arbitrary units, the positivity, respectively, of VCAM-1 (**g**) and ICAM-1 (**h**) obtained, for both adhesion molecules, evaluating fifteen non-overlapping fields with the same area for each experimental animal. The negative control of the double immunofluorescence staining without primary antibody and in the presence of isotype matched immunoglobulins G is reported in (**i**). Statistical analyses comparing multiple continuous outcomes were performed using a one-way analyses of variance test corrected by Bonferroni for immunomorphometrical evaluations. Continuous variables are summarized as means ± standard deviation. Error bars represent the 95% confidence interval around the mean; red arrows indicate the positive staining for VCAM-1; green arrows indicate the positive staining for ICAM-1; white arrows indicate the double staining for VCAM-1/ICAM-1; * indicates the level of significance, *p* ≤ 0.05; I, tunica intima; M, tunica media; A, tunica adventitia; CTR, control group; MLT, mice treated with melatonin; AU, arbitrary units.

We investigated the expression of the vascular adhesion molecule-1 (VCAM-1) and intracellular adhesion molecule-1 (ICAM-1) and considered them as general markers of the inflammatory state. The double immunofluorescence evaluation of VCAM-1 (identified in red staining in Figure 2a,d) and ICAM-1 (identified in green staining in Figure 2b,e) in the control mice showed a moderate expression of both adhesion proteins in the tunica intima (merged expression reported in Figure 2c) instead of an absent/very weak expression at the tunica intima level of ApoE^-/-^ mice treated with MLT (merged expression reported in Figure 2f).

Figure 2g,h respectively report the immunomorphometrical analyses of VCAM-1 and ICAM-1. Furthermore, Figure 2i shows the negative control of the double immunofluorescence staining.

### 2.3. Macrophage Population and Related Cytokines

After confirming the role of MLT in reducing inflammation, we wanted to assess the presence of the aortic macrophage population through the lesion and the aortic wall; in particular, we considered the expression of the pan-macrophagic marker CD68.

**Figure 3:**
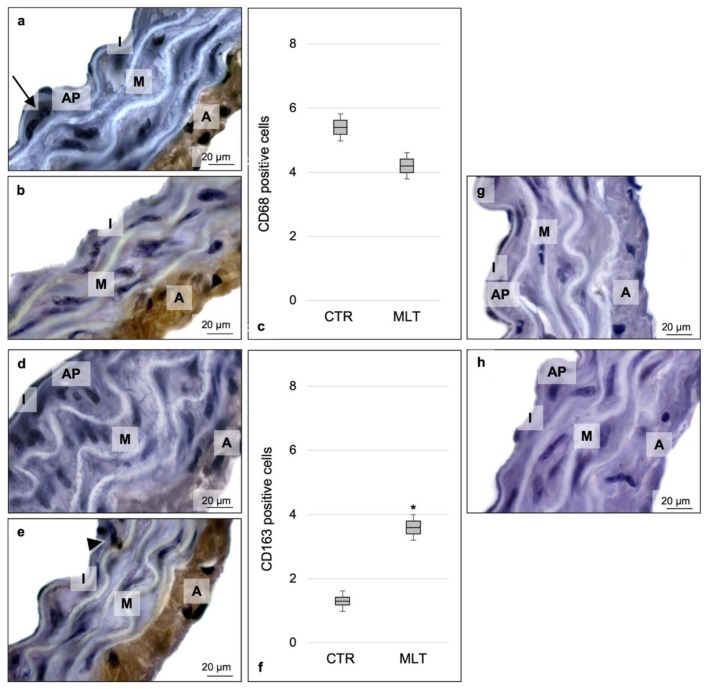
Immunohistochemical characterization of macrophage population. Labelling of the aorta’s wall with CD68 (**a**,**b**) and CD163 (**d**,**e**) antibodies. Each panel shows a full-field image at 1000× (scale bars: 20 µm). Immunohistochemical photomicrograph shows the positivity for CD68, mainly localized in the subendothelial space and in the tunica adventitia of control mice (**a**). In contrast, the immunohistochemical photomicrograph of ApoE^-/-^ mice treated with melatonin highlights that this immunopositivity slightly decreased in the subendothelial space, even if not significantly, after melatonin supplementation (**b**). CD163 expression was negative or really weak in control mice (**d**). In contrast, the photomicrograph of MLT-treated mice showed that CD163 was moderately/strongly expressed both in the subendothelial space and tunica adventitia, increasing its positivity after melatonin administration (**e**). Graphs summarize the number of CD68 (**c**) and CD163 (**f**) positive cells, obtained for both macrophage markers, evaluating five non-overlapping fields with the same area from three non-consecutive aorta sections. For both CD68 (**g**) and CD163 (**h**), sections without primary antibody and in the presence of isotype matched immunoglobulins G served as negative immunohistochemical controls. Statistical analyses comparing multiple continuous outcomes were performed using one-way analyses of variance test corrected by Bonferroni for immunomorphometrical evaluations. Continuous variables are summarized as means ± standard deviation. Error bars represent the 95% confidence interval around the mean; * indicates the level of significance, *p* ≤ 0.05; black arrow indicates CD68 positive macrophage; black arrowhead indicates CD163 positive M2 macrophage; I, tunica intima; M, tunica media; A, tunica adventitia; AP, atherosclerotic plaque; CTR, control group; MLT, mice treated with melatonin.

CD68 immunostaining identified both M1 and M2 populations and showed a weak positivity in the tunica adventitia and subendothelial space of the control mice (Figure 3a). This immunopositivity slightly decreased in the ApoE^-/-^ treated mice. CD68 expression was lower in the MLT-treated group, even if not significantly. Moreover, in this group, interestingly, CD68 expression changed its localization, being mainly evident in the aortic tunica adventitia with respect to the other morphological part of the aorta (Figure 3b).

To better characterize the macrophage population, CD163, a M2 macrophage marker, was considered. CD163 expression was negative or really weak in the control mice (Figure 3d), being, on in contrast, moderately/strongly expressed in both the subendothelial space and tunica adventitia of MLT-treated mice (Figure 3e).

Through the expression of CD68 and CD163, the presence of both M1 and M2 macrophages and, specifically, of CD163 M2 macrophages were evaluated. Then, the number of CD68 cells and CD163 macrophages were considered and are reported in Figure 3c,f, respectively.

Finally, Figure 3g,h show the CD68 and CD163 immunohistochemical negative controls, respectively.

**Figure 4:**
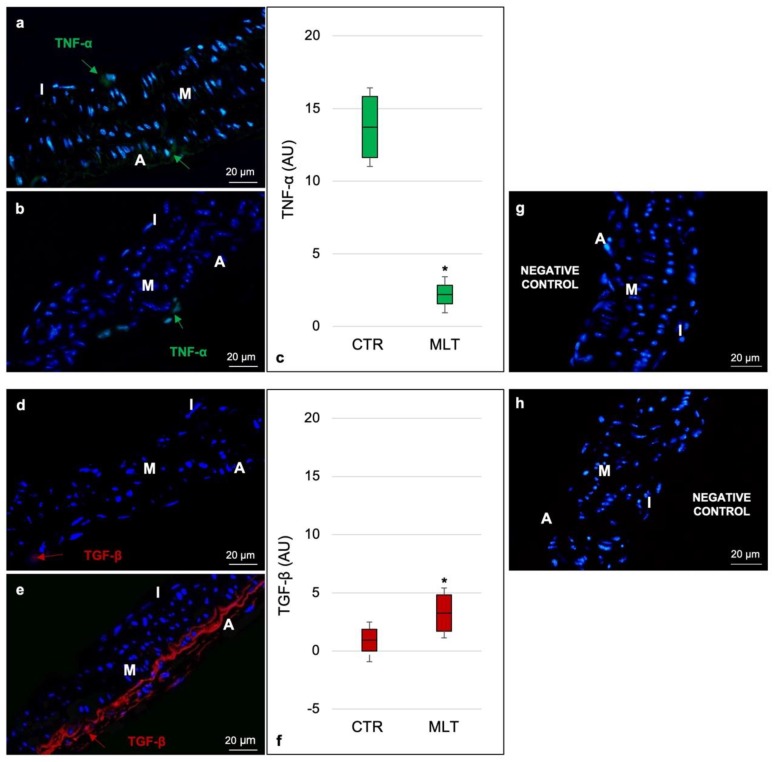
Immunofluorescence characterization of macrophage cytokine production. Labelling of the aorta’s wall with TNF-α (**a**,**b**) and TGF-β (**d**,**e**) antibodies. Each panel shows a full-field image at 400× (scale bars: 20 µm). Immunofluorescence photomicrograph of control mice showed a moderate TNF-α expression at the subendothelial and adventitia levels, confirming that these mice were characterized by an infiltrate of macrophages predominantly polarized in the M1 direction (**a**). In contrast, the immunofluorescence photomicrograph of ApoE^-/-^ mice treated with melatonin highlights, for the same M1 marker, an absent/very weak positivity (**b**). Immunofluorescence photomicrograph also showed an absent expression of TGF-β in the aorta of the control mice (**d**). Differently, the immunofluorescence photomicrograph of MLT-treated mice showed a moderate TGF-β expression in the tunica adventitia (**e**). Graphs summarize, as arbitrary units, the immunomorphometrical analyses of TNF-α (**c**) and TGF-β (**f**) obtained, for both pro-inflammatory markers, evaluating fifteen non-overlapping fields with the same area for each experimental animal, respectively. For both TNF-α (**g**) and TGF-β (**h**), sections without primary antibody and in the presence of isotype matched immunoglobulins G served as negative immunofluorescence controls. Statistical analyses comparing multiple continuous outcomes were performed using one-way analyses of variance test corrected by Bonferroni for immunomorphometrical evaluations. Continuous variables are summarized as means ± standard deviation. Error bars represent the 95% confidence interval around the mean; * indicates the level of significance, *p* ≤ 0.05; red arrows indicate the positive staining for TNF-α; green arrows indicate the positive staining for TGF-β; I, tunica intima; M, tunica media; A, tunica adventitia; CTR, control group; MLT, mice treated with melatonin; AU, arbitrary units.

To better define the macrophage’s role in atherosclerosis, we evaluated the expression of tumor necrosis factor-α (TNF-α; identified in green staining in Figure 4a,b) and of transforming growth factor-β (TGF-β; identified in green staining in Figure 4d,e), which are markers of M1 and of M2 activity, respectively.

Results confirmed that control mice were characterized by an infiltrate of macrophages predominantly polarized in the M1 direction and showed a moderate TNF-α expression at subendothelial and adventitia levels (Figure 4a). In contrast, the positivity for the same M1 marker was absent/very weak in ApoE^-/-^ mice treated with MLT (Figure 4b).

In conclusion, the TGF-β study validated what was previously shown by CD163 positivity: as a cytokine mainly associated with M2 polarization, its expression was absent in the aorta of the control mice (Figure 4d), while the MLT-treated mice showed a moderate TGF-β expression in the tunica adventitia (Figure 4e).

Figure 4c,f report the immunomorphometrical analyses of TNF-α and TGF-β, respectively.

Finally, Figure 4g,h show the TNF-α and TGF-β immunofluorescence negative controls, respectively.

### 2.4. Aortic ^18^F-FDG PET/CT Evaluation

**Figure 5:**
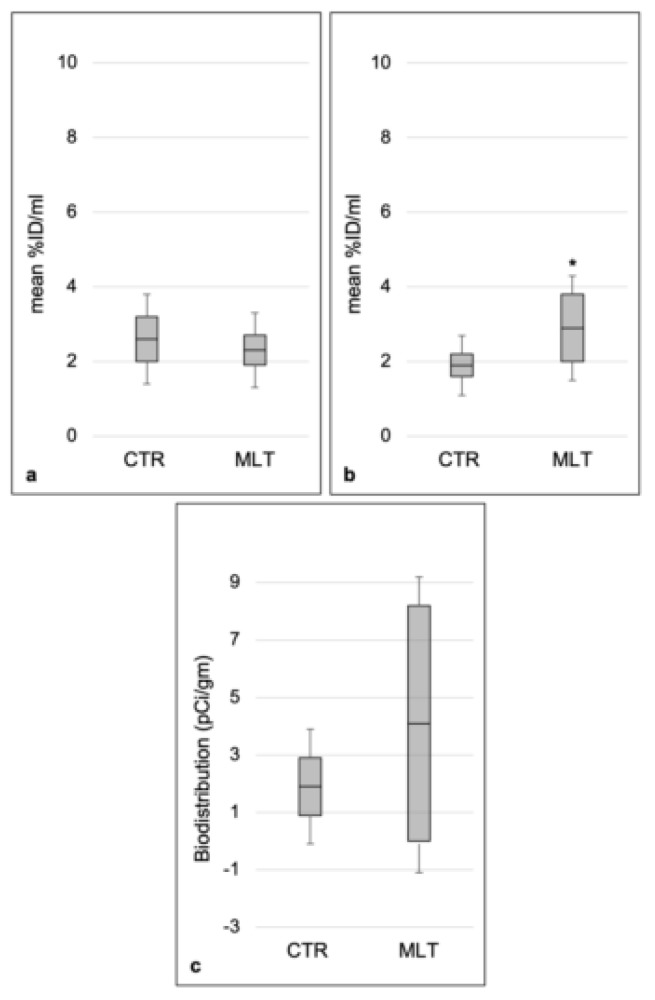
Aortic ^18^F-FDG uptake and biodistribution. Results were obtained from scans that were performed on a dedicated small animal PET/CT scanner and are expressed as mean %ID/mL (%injected dose per mL of tissue) from ^18^F-FDG PET/CT (**a**,**b**) and activity concentration (mean pCi/gm) from biodistribution data (**c**). The latter was divided by 10^5^ for the purpose of visualization. Error bars represent the 95% confidence interval around the mean; * indicates the level of significance, *p* ≤ 0.05; *CTR* control group; MLT, mice treated with melatonin; %ID/mL, % injected dose per mL of tissue; pCi/g, picocurie per gram.

On the baseline PET/CT images, mean aortic ^18^F-FDG uptake, expressed as mean %ID/mL (%injected dose per mL of tissue) was not significantly different between the control (2.6 ± 0.6 %ID/mL) and MLT-treated (2.3 ± 0.4 %ID/mL) groups (*p* = 0.5) (Figure 5a). Interestingly, after 14 weeks of atherogenic diet, follow-up PET/CT scans demonstrated significantly higher (*p* = 0.049) mean aortic ^18^F-FDG uptake in the MLT-treated group (2.9 ± 0.9 %ID/mL) compared to the control group (1.9 ± 0.3 %ID/mL) (Figure 5b).

^18^F-FDG activity concentration detected in the aorta, counted immediately after 14 weeks PET scans, showed higher values for the MLT-treated group (4 ± 4.1 × 105 pCi/gm) compared to the control group (1.9 ± 1.0 × 105 pCi/gm), even if with no statistically significant differences (*p* = 0.2) (Figure 5c).

**Figure 6:**
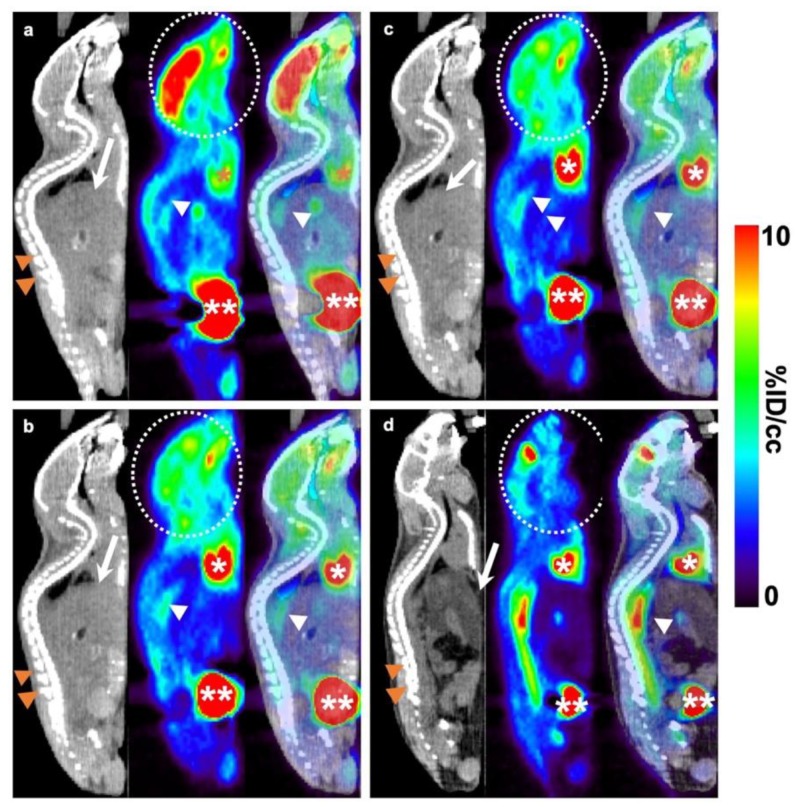
^18^F-FDG PET/CT evaluation. Sagittal views of MLT-treated mouse (**c**,**d**) and comparable views of a control mouse (**a**,**b**). Each panel shows, from left to right, CT only, PET only, and fused PET/CT images. Liver is recognized as a large solid structure in CT image and vertebral column as a hyperdense (white) posterior structure. In PET and fused PET/CT images, the sites with the most intense activity are the heart and the urinary bladder due to the normal excretion of ^18^F-FDG by the kidneys (not shown in this section). The brain may demonstrate variable levels of ^18^F-FDG uptake. The structure lying anterior to the vertebral column represents the abdominal aorta. Mild ^18^F-FDG uptake is seen in its proximal part at the baseline PET/CT images taken from the control (**a**) and MLT-treated (**c**) mice. After 14 weeks of the atherogenic diet, a second PET/CT scan showed nearly unchanged FDG activity in the control mice (**b**) while, the MLT-treated group presented a significantly higher ^18^F-FDG uptake in the same region of the aorta (**d**) compared to the baseline (respectively (**a**) for the control group and **c** for the MLT-treated mice). %ID/cc %injected dose per mL of tissue; white arrow indicates the liver; two orange arrowheads indicate the vertebral column; white asterisk indicates the heart; two white asterisks indicate the urinary bladder; white dashed circle indicates the brain; white arrowhead indicates the abdominal aorta.

The images obtained from the first scan (baseline) and after 14 weeks of atherogenic diet (follow-up) are reported in Figure 6: sagittal views of the control mouse (Figure 6a,b) were compared with those obtained from the MLT-treated mouse (Figure 6c,d). Each panel (Figure 6a–d) showed, from left to right, CT only, PET only, and fused PET/CT images, respectively. The liver can be recognized as a large solid structure in the CT image and vertebral column as a hyperdense (white) posterior structure. The heart and the urinary bladder can be identified as the sites with the most intense activity in the PET and fused PET/CT images, while the brain may demonstrate variable levels of ^18^F-FDG uptake.

The structure lying anterior to the vertebral column represents the abdominal aorta, the specific site analyzed during this study. Mild ^18^F-FDG uptake has been detected in its proximal part at the baseline PET/CT images taken from the control (Figure 6a) and MLT-treated (Figure 6c) mice. After 14 weeks of the atherogenic diet, the second PET/CT scan showed significantly higher ^18^F-FDG uptake in the same region of the aorta in the MLT-treated group (Figure 6d). In contrast, in the control group, the segment representing the abdominal aorta appeared nearly unchanged in FDG activity (Figure 6b) compared to the baseline (Figure 6a).

## 3. Discussion

In this work we provided insights into the protective role of MLT in the development of atherosclerotic process by morphological and ^18^F-FDG-PET/CT analyses.

The effects of MLT that have already been reported by many authors as well as our group [13,25,26], showed that this indoleamine is able to act on inflammation. Here, we demonstrated that ApoE^-/-^ mice treated with MLT had unremarkable morphological changes and very low atherogenic marker expression.

Of particular interest is to note that the morphological analyses showed that the ApoE^-/-^ MLT-treated mice had a higher number of M2 macrophages with respect to the M1 cells and the control animals. These animals also showed a high amount of BAT activity with respect to the control mice. These findings are in line with previous evidence reporting that the macrophage subtypes have several roles in immune response due to their innate ability to adapt to the microenvironmental changes of the host [27]. Furthermore, a compelling clinical study showed that both subtypes were evident in human plaques, but M2 macrophages were localized in a stable position inside the lesion, and the expression of their markers was inversely related with disease progression. M1 macrophages were abundant in vulnerable plaques whereas M2 cells were fewer in the atherosclerosis animal model. Both of these populations are plastic cells because they can switch from the M1 to M2 state and vice versa upon specific signals; the changes from M1 to M2 has been defined as “macrophage polarization” [28]. Furthermore, M1 macrophages exhibit elevated lipid accumulation, which contribute to the acceleration of atherosclerosis; whereas in contrast, M2 macrophages are located far from the lipid core of the plaque, contain smaller lipid droplets, and are involved in phagocytosis [29,30,31]. In particular, Chinetti et al. showed that in the adventitia, M2 macrophages are twofold to threefold more abundant than M1 macrophages [29]. Moreover, it has been shown that in ApoE^-/-^ mice, the major site of vascular inflammatory cell accumulation is adventitia rather than the intima, and in atherosclerotic human aorta, inflammatory cells were observed to be present in perivascular adipose tissue at the adventitial margin [32,33]. Furthermore, Moos et al. demonstrated that in atherosclerotic arteries, the lamina adventitia is a major compartment of wall inflammation with lymphocyte infiltration and lymphoid follicle-like organogenesis [32]. Therefore, it is possible that the increase of M2 macrophages, stimulated by MLT, is necessary for reducing the atherosclerotic process. This hypothesis could be linked to the functions of these cells, which are related to anti-inflammatory expression markers, as reported by Yang et al. [34]. To support this hypothesis, some researchers have shown that MLT ameliorates inflammation by suppressing the cells toward the pro-inflammatory M1 phenotype and circadian nuclear receptor.

WAT mass or activity is positively correlated with the development of some diseases (e.g., obesity and type 2 diabetes) because it stores surfeit lipid, rendering the macromolecules in adipocytes particularly vulnerable to carbonylation and other modifications driven by oxidative stress. On the contrary, BAT is negatively correlated with the diseases reported above; it is a metabolically active fat with both local and systemic antiatherogenic effects [35,36,37]. Furthermore, it is known that BAT metabolism daily and seasonal variations are regulated by MLT through not well-known mechanisms [38,39]. Our data indicate that the aorta of the atherosclerotic mice model had more periaortic WAT with respect to the same animals treated with MLT. Instead, these animals showed a greater amount of periaortic BAT.

All data reported above corroborate our findings suggesting that MLT plays an important role in the reduction/suppression of the atherosclerotic process; this indoleamine is able to stimulate M2 macrophages, the production of their cytokines, and preserve periaortic BAT activity, which is crucial for organism homeostasis [39].

Another important finding of this study is the possibility of extending the vasoprotective effects of MLT on atherosclerosis, showing that ^18^F-FDG can be used to measure the progression of the atherosclerotic process as well as measure the periaortic BAT activity. The evidence to support this interpretation is not complete [22]. Thereby, detailed studies in this direction could be the key for demonstrating the capacity of the ^18^F-FDG marker and stressing the benefits of MLT with an easy technical approach.

Our findings suggest that ^18^F-FDG uptake is very high in mice treated with MLT and these data do not seem to be in line with the morphological data on atherosclerotic progression. Therefore, aimed at this purpose, we wanted to consider, first at all, the diagnostical characteristics of the ^18^F-FDG marker and, second, compare the morphological results with clinical data.

Several studies have shown that ^18^F-FDG is used as a diagnostic marker to reveal physiological or pathological glucose metabolism changes at the molecular level [40]. This agent has sensitivity and accuracy for tumor diagnosis, staging, and treatment response [41]. Moreover, some researchers have studied the ^18^F-FDG marker in normal tissues and some factors that can influence its uptake such as age, gender, and blood glucose [42,43].

However, it is possible that other factors that may alter ^18^F-FDG distribution are not well-known [39]. In this context, our study supports the last hypothesis; in fact, we showed that ^18^F-FDG uptake is very weak in the atherosclerotic mice model, but high in the same animals treated with MLT. The focus on these findings could be linked to the increased number of M2 macrophages and to the high activity of BAT, which have been seen by morphological analyses. Therefore, the presence of M2 macrophages and periaortic BAT can be other factors involved in ^18^F-FDG uptake. Na and Choi proposed that the M2 macrophage enrichment score was positively correlated to ^18^F-FDG uptake in advanced head and neck squamous cell carcinoma [44]. According to these authors, the competition for glucose between cancer and immune cells plays an important role in the tumor progression associated with hypermetabolic characteristics.

Regarding periaortic BAT activity, several studies have suggested a relation between total volume of activated BAT, evaluated by ^18^F-FDG-PET/CT, and a positive prognostic factor of tumor [45,46]. They demonstrated that ^18^F-FDG uptake was due to periaortic BAT metabolic activity, which interferes with oncological conditions. From this point of view, our observations obtained by ^18^F-FDG marker were consistent with the studies reported above and showed a positive network with morphological analyses. Moreover, there have been many experimental studies reporting that this method is used to evaluate atherosclerosis in mouse atherosclerosis models [47,48]. These data raise questions about the use of ^18^F-FDG-PET/CT in ApoE^-/-^ mice, indicating that periaortic adipose tissue is a confounding factor for atherosclerosis. Our data were in agreement with these results, showing that ^18^F-FDG uptake is linked to adipose tissue. MLT increases periaortic BAT and this confirms the evidence of the benefits conferred by the stimulation of this tissue, giving a further demonstration of its possible therapeutic role as reported by Oliver et al. [49].

In conclusion, the present study demonstrated that MLT could be considered a “therapeutic” strategy for atherosclerosis, but the very important findings regard the possibility of using ^18^F-FDG-PET/CT for monitoring and/or management of non-neoplastic applications.

Last but not least, our study stresses how two different approaches such as morphological and imaging data lead to the same results, suggesting that ^18^F-FDG is a sensitive marker even for marking changes associated with the homeostasis of the organism. In this case, the marker also seems to be, accurate for elucidating the beneficial effects of MLT in atherosclerotic processes by demonstrating increased periaortic BAT mass and M2 polarization.

## 4. Materials and Methods

### 4.1. Animals and Treatments

All animal experiments were conducted upon approval (17 December 2015) of the University of California, San Francisco (UCSF) Institutional Animal Care and Use Committee (IACUC) and supervision of the Laboratory Animal Research Center (LARC), which is accredited by the Association for Assessment and Accreditation of Laboratory Animal Care International (AAALAC International).

A total of 20 ApoE^-/-^ mice (JAX B6.129P2-Apoetm1Unc/J, strain #003000, The Jackson Laboratory, Bar Harbor, ME, USA) were studied. Since the 4th week of life, all mice were treated with an atherogenic high-fat “Western” diet (0.2% total cholesterol, saturated fat >60% total fat and high sucrose; TD 88137, Harlan Teklad, Harlan Laboratories, Madison, WI, USA) for the entire duration of the study (14 weeks) to expedite the development of atherosclerotic process; all mice had free access to food. When the atherogenic diet started, mice were randomly assigned to two different groups: the first group (10 mice) was treated with MLT (Melapure™, Flamma S.p.A., Chignolo d’Isola – BG, Italy) dissolved in a minimum volume of ethanol (0.7–0.9% of the final solution) and diluted in drinking water to yield the final dose of 10 mg/kg body weight/day. The dose selection was based on previous studies, showing a preventive effect of MLT administration on endothelial dysfunction at a dose level of 10 mg/kg/day [50,51]. The second group (10 mice) was treated orally with drinking water plus vehicle of MLT (ethanol). The sample size was obtained through the analyses of the factorial variance with a fixed effect model and to reach the power of 0.82 at a significance of 0.05, 10 animals per experimental group were used.

Each mouse was identified by a unique ear-notch punch without a metal ear tag in order to avoid a metal artifact in CT; in addition, mice were monitored daily for wounds or signs of distress and weekly for weight changes. All mice were euthanized at the end of the study by anesthetic overdose (5% isoflurane) followed by cervical dislocation, conforming to the standard established by the UCSF LARC.

### 4.2. Histopathological Analyses

Aorta specimens from the abdominal tract were rinsed in physiological solution and used for the morphometrical, immunohistochemical, and immunofluorescences analyses. They were fixed in 4% buffered paraformaldehyde for 24 h, dehydrated in graded ethanol, and then embedded in paraffin wax, following the standard protocol [50,52]. Serial paraffin sections (7 μm thick) of each sample were cut with a microtome.

### 4.3. Morphometrical Analyses

Alternate paraffin sections were dewaxed, rehydrated, and stained with hematoxylin-eosin, following the standard protocol. Fifteen non-overlapping fields with the same area (0.04 µm^2^/field) and randomly selected for each experimental animal were observed with an optical light microscope (Olympus, Hamburg, Germany) in order to evaluate periaortic BAT and WAT. In particular, brown adipocytes have been recognized thanks to their characteristic multilocularity and abundance of big typical mitochondria. Moreover, they are morphologically and functionally different from white adipocytes: BAT is mainly composed of small, polygonal cells with ~50% of the volume occupied by lipids partitioned into several droplets, on the contrary, the parenchyma of WAT is composed of unilocular, lipid-laden white adipocytes, characterized by only few and small mitochondria and by few sympathetic noradrenergic nerve fibers [24,53].

Histological whole-slide images were obtained at the magnitude of 400 and 1000 times the original size, using a QImaging QICAM digital camera (High-Performance IEEE 1394 FireWire™ Digital CCD Camera, 1.4 million pixels, 12-bit digital output, Surrey BC, Canada V3S 6K3) and an optical light microscope (Olympus, Hamburg, Germany) application supplied by the manufacturers.

The percentage per area of periaortic BAT and WAT was calculated using computerized image analyzing software (Image Pro Premier 9.1, MediaCybernetics, Rockville, MD, USA).

Two blinded investigators performed the morphometrical analyses using an image analyzer; in the case of dispute concerning interpretation, the case was reviewed to reach an agreement.

### 4.4. Immunofluorescence, Immunohistochemistry, and Immunomorphometrical Analyses

Alternate paraffin sections were dewaxed, rehydrated, and incubated in 3% hydrogen peroxide for 30 min. Then, after the blocking step in 3% bovine serum albumin solution for 1 h, the sections were incubated 1 h at 37 °C and 30 min at room temperature with the following primary antibodies: rat monoclonal antibody against CD68 (diluted 1:100; Abcam, Cambridge, UK); rabbit polyclonal antibody against CD163 (diluted 1:50; Abcam, Cambridge, UK); goat polyclonal antibody against TNF-α (diluted 1:200 Santa Cruz Biotechnology Inc., Dallas, TX, USA) [54]; mouse monoclonal antibody against TGF-β (diluted 1:150; Santa Cruz Biotechnology Inc., Dallas, TX, USA) [55] and simultaneously with mouse monoclonal antibody against ICAM-1 (diluted 1:200; Santa Cruz Biotechnology Inc., Dallas, TX, USA) and rabbit polyclonal antibody against VCAM-1 (diluted 1:200; Santa Cruz Biotechnology Inc., Dallas, TX, USA). For immunofluorescence analyses of TNF-α, TGF-β, ICAM-1, and VCAM-1, the sections were labelled with specific conjugated secondary antibodies (diluted 1:200; Invitrogen, Paisley, UK), counterstained with 4′,6-diamidino-2-phenylindole (DAPI), mounted, and observed with a fluorescent microscope (Nikon, Düsseldorf, Germany) with red/green/blue filters at final magnification of 400× [56,57,58].

For immunohistochemical analyses of CD68 and CD163, the aorta sections were sequentially incubated in specific biotinylated immunoglobulins and in avidin-biotin peroxidase complex. The reaction products were visualized using 0.33% hydrogen peroxide and 0.05% 3,3′-diaminobenzidine tetrahydrochloride as the chromogen. The sections were finally counterstained with hematoxylin, mounted, and observed with a light microscope (Olympus, Hamburg, Germany) at a final magnification of 1000×.

Sections without primary antibody and in the presence of isotype matched immunoglobulins G served as negative immunofluorescent/immunohistochemical controls. In detail, the double immunofluorescence negative control was obtained with a mixed solution of secondary antibodies.

Fifteen non-overlapping fields with the same area (0.04 µm^2^/field) and randomly selected for each experimental animal were analyzed and the immunopositivity for each primary antibody was calculated using a computerized image analyzer software (Image Pro Premier 9.1, Media Cybernetics, Rockville, MD, USA). The immunopositivity for each primary antibody was calculated as integrated optical density (IOD) automatically compared to the area considered. The IOD value reports the average intensity/density of each stained region and it was expressed by the software as arbitrary units (AU) [59,60]. Furthermore, the number of CD68 and CD163 positive cells were counted in randomly selected five non-overlapping fields with the same area (0.04 µm^2^/field) from three non-consecutive aorta sections for each experimental animal [59,60]. Two blinded investigators performed the immunomorphometrical analyses and, again, in case of dispute concerning interpretation, the case was reviewed to reach an agreement.

### 4.5. ^18^F-FDG PET Imaging and Biodistribution

Mice were fasted with ad libitum access to beverage at least for 6 h before receiving 10.1 ± 0.45 MBq/kg of FDG in 0.1 mL volume via tail vein. All mice were imaged at the baseline (the day before starting the atherogenic diet) and after 14 weeks of the atherogenic diet.

Twenty mice completed ^18^F-FDG PET/CT scans at the baseline but only nineteen completed ^18^F-FDG PET/CT scans at the follow-up time point.

The PET data were acquired 60 min after radiotracer administration with a single 20-min bed position, followed by CT for attenuation correction of PET reconstruction. Animals were kept at room temperature during uptake time and scan, and under anesthesia with 2% isoflurane mixed with medical grade oxygen.

All scans were performed on a dedicated small animal PET/CT scanner (Inveon, Siemens Healthcare, Malvern, PA, USA). PET images were reconstructed using the ordered subsets expectation maximization (OSEM) algorithm, provided by the manufacturer. The resulting PET images had a 128 × 128 × 159 matrix with a voxel size of 0.776 × 0.776 × 0.796 mm^3^. CT images were reconstructed using a cone-beam Feldkamp reconstruction algorithm (COBRA, Exxim Computing Corporation, Pleasanton, CA, USA). The reconstructed CT images had a 512 × 512 × 662 matrix size with an isotropic voxel size of 0.191 mm^3^. The coregistered attenuation map from CT, obtained via a pre-derived rigid transformation matrix, was used for attenuation correction of the PET data.

Following each scan, investigators monitored the animals carefully until they fully recovered from anesthesia before being held in the animal vivarium again. The experimental protocol of the research project was designed to provide the minimum level of pain or suffering, consistent with the objectives of the project.

Aorta images obtained at each time point were reviewed, then the 3D freehand tool in Amide software [61] was used to delineate volumes of interest (VOIs) on the aorta, on axial slices. Percent-injected dose per milliliter (%ID/mL) was used to quantify the FDG measurements. Both maximum and mean values were obtained and averaged over the delineated VOIs.

Furthermore, biodistribution studies were conducted immediately after the 14-week scan. After euthanizing the animals, blood samples were obtained by cardiac puncture. Thirteen samples (including the aorta, heart, lung, liver, stomach, small intestine, large intestine, pancreas, spleen, kidney, muscle, brain, and bone) were removed, rinsed in water, and dried in air for 5 min. The samples were then weighed and counted on a gamma-counter (1470 WIZARD Gamma Counter, Wallac, Finland) for accumulation of ^18^F-FDG. The uptake of radiotracer in tissues was expressed in counts per minute corrected for decay and background and expressed as picocurie per gram (pCi/g).

### 4.6. Statistical Analyses

Statistical analyses were performed with SPSS (Version 22.0, IBM Corp., Armonk, NY, USA) using a two-sided 0.05 level of significance. Student’s t-test (for numerical and approximately normally distributed data) and Mann–Whitney U tests (for numerical and not normally distributed data) were used to evaluate differences in the animal’s characteristics between the MLT-treated and control groups. Statistical analyses comparing multiple continuous outcomes were performed using the one-way analyses of variance test corrected by Bonferroni for morphometrical and immunomorphometrical evaluations. Continuous variables were summarized as means ± standard deviation.

## Abbreviations

CVDs	Cardiovascular diseases
TNF-α	Tumor necrosis factor-α
TGF-β	Transforming growth factor-β
WAT	White adipose tissue
BAT	Brown adipose tissue
MLT	Melatonin
^18^F-FDG	^18^F- fluorodeoxyglucose
PET	Positron emission tomography
CT	Computed tomography
UCSF	University of California, San Francisco
IACUC	Institutional Animal Care and Use Committee
LARC	Laboratory Animal Research Center
AAALAC	Association for Assessment and Accreditation of Laboratory Animal Care
ApoE^-/-^	Apolipoprotein-E knockout
OSEM	Ordered subset expectation maximization
VOIs	Volumes of interest
%ID/ml	%injected dose per ml of tissue
pCi/g	Picocurie per gram
CD68	Cluster of Differentiation 68
CD163	Cluster of Differentiation 163
ICAM-1	Intracellular adhesion molecule-1
VCAM-1	Vascular cellular adhesion molecule-1
DAPI	4′,6-diamidino-2-phenylindole
AU	Arbitrary units

## References

[1] Ridker P.M., Everett B.M., Thuren T., MacFadyen J.G., Chang W.H., Ballantyne C., Fonseca F., Nicolau J., Koenig W., Anker S.D. (2017). Antiinflammatory Therapy with Canakinumab for Atherosclerotic Disease. N. Engl. J. Med..

[2] Nahrendorf M., Swirski F.K. (2015). Lifestyle effects on hematopoiesis and atherosclerosis. Circ. Res..

[3] Chait A., den Hartigh L.J. (2020). Adipose Tissue Distribution, Inflammation and Its Metabolic Consequences, Including Diabetes and Cardiovascular Disease. Front. Cardiovasc. Med..

[4] Raggi P., Genest J., Giles J.T., Rayner K.J., Dwivedi G., Beanlands R.S., Gupta M. (2018). Role of inflammation in the pathogenesis of atherosclerosis and therapeutic interventions. Atherosclerosis.

[5] Moriya J. (2019). Critical roles of inflammation in atherosclerosis. J. Cardiol..

[6] Ding S., Lin N., Sheng X., Zhao Y., Su Y., Xu L., Tong R., Yan Y., Fu Y., He J. (2019). Melatonin stabilizes rupture-prone vulnerable plaques via regulating macrophage polarization in a nuclear circadian receptor RORα-dependent manner. J. Pineal. Res..

[7] Barrett T.J. (2020). Macrophages in Atherosclerosis Regression. Arterioscler. Thromb. Vasc. Biol..

[8] Shapouri-Moghaddam A., Mohammadian S., Vazini H., Taghadosi M., Esmaeili S.A., Mardani F., Seifi B., Mohammadi A., Afshari J.T., Sahebkar A. (2018). Macrophage plasticity, polarization, and function in health and disease. J. Cell. Physiol..

[9] Queiroz M., Sena C.M. (2020). Perivascular adipose tissue in age-related vascular disease. Aging Res. Rev..

[10] Goldberg I.J., Sharma G., Fisher E.A. (2020). Atherosclerosis: Making a U Turn. Annu. Rev. Med..

[11] Tan D.X., Hardeland R., Manchester L.C., Korkmaz A., Ma S., Rosales-Corral S., Reiter R.J. (2012). Functional roles of melatonin in plants, and perspectives in nutritional and agricultural science. J. Exp. Bot..

[12] Meng X., Li Y., Li S., Zhou Y., Gan R.Y., Xu D.P., Li H.B. (2017). Dietary Sources and Bioactivities of Melatonin. Nutrients.

[13] Li H., Li J., Jiang X., Liu S., Liu Y., Chen W., Yang J., Zhang C., Zhang W. (2019). Melatonin enhances atherosclerotic plaque stability by inducing prolyl-4-hydroxylase α1 expression. J. Hypertens..

[14] Favero G., Franceschetti L., Buffoli B., Moghadasian M.H., Reiter R.J., Rodella L.F., Rezzani R. (2017). Melatonin: Protection against age-related cardiac pathology. Aging Res. Rev..

[15] Favero G., Franceschetti L., Bonomini F., Rodella L.F., Rezzani R. (2017). Melatonin as an Anti-Inflammatory Agent Modulating Inflammasome Activation. Int. J. Endocrinol..

[16] Hu J., Zhang L., Yang Y., Guo Y., Fan Y., Zhang M., Man W., Gao E., Hu W., Reiter R.J. (2017). Melatonin alleviates postinfarction cardiac remodeling and dysfunction by inhibiting Mst1. J. Pineal Res..

[17] Bonomini F., Borsani E., Favero G., Rodella L.F., Rezzani R. (2018). Dietary Melatonin Supplementation Could Be a Promising Preventing/Therapeutic Approach for a Variety of Liver Diseases. Nutrients.

[18] Hardeland R. (2018). Melatonin and inflammation-Story of a double-edged blade. J. Pineal Res..

[19] Høilund-Carlsen P.F., Sturek M., Alavi A., Gerke O. (2019). Atherosclerosis imaging with F-sodium fluoride PET: State-of-the-art review. Eur. J. Nucl. Med. Mol. Imag..

[20] Dunphy M.P., Freiman A., Larson S.M., Strauss H.W. (2005). Association of vascular 18F-FDG uptake with vascular calcification. J. Nucl. Med..

[21] Rudd J.H., Myers K.S., Bansilal S., Machac J., Pinto C.A., Tong C., Rafique A., Hargeaves R., Farkouh M., Fuster V. (2008). Atherosclerosis inflammation imaging with 18F-FDG PET: Carotid, iliac, and femoral uptake reproducibility, quantification methods, and recommendations. J. Nucl. Med..

[22] Al-Mashhadi R.H., Tolbod L.P., Bloch L.Ø., Bjørklund M.M., Nasr Z.P., Al-Mashhadi Z., Winterdahl M., Frøkiær J., Falk E., Bentzon J.F. (2019). Fluorodeoxyglucose Accumulation in Arterial Tissues Determined by PET Signal Analysis. J. Am. Coll. Cardiol..

[23] Ohashi T., Terasawa K., Aoki M., Akazawa T., Shibata H., Kuze B., Asano T., Kato H., Miyazaki T., Matsuo M. (2020). The importance of FDG-PET/CT parameters for the assessment of the immune status in advanced HNSCC. Auris. Nasus. Larynx..

[24] Manieri M., Murano I., Fianchini A., Brunelli A., Cinti S. (2010). Morphological and immunohistochemical features of brown adipocytes and preadipocytes in a case of human hibernoma. Nutr. Metab. Cardiovasc. Dis..

[25] Favero G., Rodella L.F., Reiter R.J., Rezzani R. (2014). Melatonin and its atheroprotective effects: A review. Mol. Cell. Endocrinol..

[26] Li H.Y., Leu Y.L., Wu Y.C., Wang S.H. (2019). Melatonin Inhibits In Vitro Smooth Muscle Cell Inflammation and Proliferation and Atherosclerosis in Apolipoprotein E-Deficient Mice. J. Agric. Food. Chem..

[27] Gong M., Zhuo X., Ma A. (2017). STAT6 Upregulation Promotes M2 Macrophage Polarization to Suppress Atherosclerosis. Med. Sci. Monit. Basic. Res..

[28] de Gaetano M., Crean D., Barry M., Belton O. (2016). M1- and M2-Type Macrophage Responses Are Predictive of Adverse Outcomes in Human Atherosclerosis. Front. Immunol..

[29] Chinetti-Gbaguidi G., Baron M., Bouhlel M.A., Vanhoutte J., Copin C., Sebti Y., Derudas B., Mayi T., Bories G., Tailleux A. (2011). Human atherosclerotic plaque alternative macrophages display low cholesterol handling but high phagocytosis because of distinct activities of the PPARγ and LXRα pathways. Circ. Res..

[30] Chinetti-Gbaguidi G., Colin S., Staels B. (2015). Macrophage subsets in atherosclerosis. Nat. Rev. Cardiol..

[31] Bi Y., Chen J., Hu F., Liu J., Li M., Zhao L. (2019). M2 Macrophages as a Potential Target for Antiatherosclerosis Treatment. Neural. Plast..

[32] Moos M.P., John N., Gräbner R., Nossmann S., Günther B., Vollandt R., Funk C.D., Kaiser B., Habenicht A.J. (2005). The lamina adventitia is the major site of immune cell accumulation in standard chow-fed apolipoprotein E-deficient mice. Arterioscler. Thromb. Vasc. Biol..

[33] Ahmadieh S., Kim H.W., Weintraub N.L. (2020). Potential role of perivascular adipose tissue in modulating atherosclerosis. Clin. Sci..

[34] Yang S., Yuan H.Q., Hao Y.M., Ren Z., Qu S.L., Liu L.S., Wei D.H., Tang Z.H., Zhang J.F., Jiang Z.S. (2020). Macrophage polarization in atherosclerosis. Clin. Chim. Acta..

[35] Masschelin P.M., Cox A.R., Chernis N., Hartig S.M. (2020). The Impact of Oxidative Stress on Adipose Tissue Energy Balance. Front. Physiol..

[36] You M., Fan R., Kim J., Shin S.H., Chung S. (2020). Alpha-Linolenic Acid-Enriched Butter Promotes Fatty Acid Remodeling and Thermogenic Activation in the Brown Adipose Tissue. Nutrients.

[37] Raiko J., Orava J., Savisto N., Virtanen K.A. (2020). High Brown Fat Activity Correlates with Cardiovascular Risk Factor Levels Cross-Sectionally and Subclinical Atherosclerosis at 5-Year Follow-up. Arterioscler. Thromb. Vasc. Biol..

[38] Jiménez-Aranda A., Fernández-Vázquez G., Campos D., Tassi M., Velasco-Perez L., Tan D.X., Reiter R.J., Agil A. (2013). Melatonin induces browning of inguinal white adipose tissue in Zucker diabetic fatty rats. J. Pineal Res..

[39] De Souza C.A.P., Gallo C.C., de Camargo L.S., de Carvalho P.V.V., Olesçuck I.F., Macedo F., da Cunha F.M., Cipolla-Neto J., do Amaral F.G. (2019). Melatonin multiple effects on brown adipose tissue molecular machinery. J. Pineal. Res..

[40] Yao Y., Wang Y., Zhang Y., Li Y., Sheng Z., Wen S., Ma G., Liu N., Fang F., Teng G.J. (2012). In vivo imaging of macrophages during the early stages of abdominal aortic aneurysm using high resolution MRI in ApoE mice. PLoS ONE.

[41] Blodgett T.M., Meltzer C.C., Townsend D.W. (2007). PET/CT: Form and function. Radiology.

[42] Kim I.J., Kim S.J., Kim Y.K. (2009). Age- and sex-associated changes in cerebral glucose metabolism in normal healthy subjects: Statistical parametric mapping analysis of F-18 fluorodeoxyglucose brain positron emission tomography. Acta. Radiol..

[43] Büsing K.A., Schönberg S.O., Brade J., Wasser K. (2013). Impact of blood glucose, diabetes, insulin, and obesity on standardized uptake values in tumors and healthy organs on 18F-FDG PET/CT. Nucl. Med. Biol..

[44] Na K.J., Choi H. (2018). Tumor Metabolic Features Identified by F-FDG PET Correlate with Gene Networks of Immune Cell Microenvironment in Head and Neck Cancer. J. Nucl. Med..

[45] Hany T.F., Gharehpapagh E., Kamel E.M., Buck A., Himms-Hagen J., von Schulthess G.K. (2002). Brown adipose tissue: A factor to consider in symmetrical tracer uptake in the neck and upper chest region. Eur. J. Nucl. Med. Mol. Imaging.

[46] Pace L., Nicolai E., Basso L., Garbino N., Soricelli A., Salvatore M. (2020). Brown Adipose Tissue in Breast Cancer Evaluated by (F) FDG-PET/CT. Mol. Imaging. Biol..

[47] Laurberg J.M., Olsen A.K., Hansen S.B., Bottcher M., Morrison M., Ricketts S.A., Falk E. (2007). Imaging of vulnerable atherosclerotic plaques with FDG-microPET: No FDG accumulation. Atherosclerosis.

[48] Toczek J., Broisat A., Perret P., Desruet M.D., Fagret D., Riou L.M., Ghezzi C. (2014). Periaortic brown adipose tissue as a major determinant of (¹⁸F)-fluorodeoxyglucose vascular uptake in atherosclerosis-prone, apoE^-/-^ mice. PLoS ONE.

[49] Oliver P., Lombardi A., De Matteis R. (2020). Editorial: Insights into Brown Adipose Tissue Functions and Browning Phenomenon. Front. Physiol..

[50] Rezzani R., Favero G., Stacchiotti A., Rodella L.F. (2013). Endothelial and vascular smooth muscle cell dysfunction mediated by cyclophylin A and the atheroprotective effects of melatonin. Life Sci..

[51] Rodella L.F., Favero G., Foglio E., Rossini C., Castrezzati S., Lonati C., Rezzani R. (2013). Vascular endothelial cells and dysfunctions: Role of melatonin. Front. Biosci..

[52] Favero G., Stacchiotti A., Castrezzati S., Bonomini F., Albanese M., Rezzani R., Rodella L.F. (2015). Melatonin reduces obesity and restores adipokine patterns and metabolism in obese (ob/ob) mice. Nutr. Res..

[53] Giordano A., Frontini A., Cinti S. (2016). Convertible visceral fat as a therapeutic target to curb obesity. Nat. Rev. Drug. Discov..

[54] Bonomini F., Taurone S., Parnigotto P., Zamai L., Rodella L.F., Artico M., Rezzani R. (2016). Role of parnaparin in atherosclerosis. Int. J. Exp. Pathol..

[55] Rodella L.F., Rossini C., Favero G., Foglio E., Loreto C., Rezzani R. (2012). Nicotine-induced morphological changes in rat aorta: The protective role of melatonin. Cells Tissues Organs.

[56] Favero G., Trapletti V., Bonomini F., Stacchiotti A., Lavazza A., Rodella L.F., Rezzani R. (2017). Oral Supplementation of Melatonin Protects against Fibromyalgia-Related Skeletal Muscle Alterations in Reserpine-Induced Myalgia Rats. Int. J. Mol. Sci..

[57] Oliveira V.A., Favero G., Stacchiotti A., Giugno L., Buffoli B., de Oliveira C.S., Lavazza A., Albanese M., Rodella L.F., Pereira M.E. (2017). Acute mercury exposition of virgin, pregnant, and lactating rats: Histopathological kidney and liver evaluations. Environ. Toxicol..

[58] Favero G., Paini A., De Ciuceis C., Rodella L.F., Moretti E., Porteri E., Rossini C., Ministrini S., Solaini L., Stefano C. (2018). Changes in extracellular matrix in subcutaneous small resistance arteries of patients with essential hypertension. Blood Press..

[59] Agabiti-Rosei C., Favero G., De Ciuceis C., Rossini C., Porteri E., Rodella L.F., Franceschetti L., Maria Sarkar A., Agabiti-Rosei E., Rizzoni D. (2017). Effect of long-term treatment with melatonin on vascular markers of oxidative stress/inflammation and on the anticontractile activity of perivascular fat in aging mice. Hypertens. Res..

[60] Bonomini F., Favero G., Rodella L.F., Moghadasian M.H., Rezzani R. (2018). Melatonin modulation of sirtuin-1 attenuates liver injury in a hypercholesterolemic mouse model. Biomed. Res. Int..

[61] Loening A.M., Gambhir S.S. (2003). AMIDE: A free software tool for multimodality medical image analysis. Mol. Imaging.

